# Body mass index and prostate cancer incidence: a comprehensive systematic review

**DOI:** 10.3389/fonc.2026.1766743

**Published:** 2026-02-25

**Authors:** Dawson Myers, Daniela Chissum Lagos, Isain Zapata, Clara Hwang

**Affiliations:** 1 Rocky Vista University, College of Osteopathic Medicine, Englewood, CO, United States; 2 Department of Biomedical Sciences, Rocky Vista University, Englewood, CO, United States; 3 Office of Research & Scholarly Activity, Rocky Vista University, Englewood, CO, United States; 4 Henry Ford Cancer Institute, Henry Ford Hospital, Detroit, MI, United States

**Keywords:** BMI, cancer screening, metabolic dysregulation, obesity, prostate cancer

## Abstract

**Introduction:**

Prostate cancer, the second leading cause of cancer-related mortality among men in the U.S., is projected to cause over 36,000 deaths in 2025. This systematic literature review investigates the association between body mass index (BMI) and prostate cancer incidence, evaluating evidence from 2004 to October 2025.

**Methods:**

A comprehensive search of PubMed using the keywords “BMI” and “prostate cancer” and “screening” identified 60 articles, from which 24 relevant studies were selected for direct analysis.

**Results:**

While some studies show a positive correlation between higher BMI and increased risk of prostate cancer, others report no significant association or an inverse relationship. Thus, the overall evidence supports a neutral relationship between BMI and prostate cancer risk.

**Discussion:**

The review explores potential biological mechanisms linking BMI to prostate cancer, including metabolic dysregulation, hormonal changes, and genetic factors. Additionally, it examines how factors such as age, race/ethnicity, and socioeconomic status may modify this relationship. The review highlights that although a higher BMI is generally associated with increased prostate cancer risk and poorer outcomes, the evidence is inconsistent. Key limitations include high variability in study designs and outcome measures, short follow-up periods as well as studies predominantly from Western populations. These inconsistencies underscore the need for further research to clarify the relationship and improve targeted interventions to mitigate prostate cancer’s impact.

## Introduction

Prostate cancer is the second leading cause of cancer-related mortality among men in the United States. In 2025, the American Cancer Society projects approximately 35,770 deaths, underscoring the public health burden of this disease. Globally, prostate cancer remains a significant health concern, with incidence rates varying widely across different ages, ethnic groups, and socioeconomic contexts.

The etiology of prostate cancer involves many factors, including genetic predisposition, environmental influences, and lifestyle choices. Key risk factors such as age, family history, race, and diet have been well established. However, the impact of modifiable factors like Body Mass Index (BMI) on prostate cancer risk is less conclusive, and findings in the literature are inconsistent. Some studies suggest that elevated BMI (>25 is overweight) may increase the risk of prostate cancer, while others report no significant association or even a protective effect against early-stage disease (1, 2). This ambiguity creates challenges for clinicians, researchers, and policymakers aiming to develop evidence-based prevention strategies.

The current knowledge gap revolves around understanding the nuanced relationship between BMI and prostate cancer incidence, progression, and mortality. Although metabolic and hormonal disruptions associated with obesity (such as insulin resistance, chronic inflammation, and altered testosterone levels) are hypothesized to contribute to prostate carcinogenesis, the precise pathways remain poorly defined. Additionally, inconsistencies in study methodologies—such as variations in BMI measurement, population demographics, and follow-up duration—further complicate the interpretation of existing evidence.

This literature review aims to explore the relationship between BMI and prostate cancer incidence from a population health perspective, with a focus on identifying evidence-based trends, potential modifiers, and gaps in current research. We hypothesize that BMI-associated metabolic alterations promote a biological environment favorable to cancer cell proliferation and thereby contribute to the development of prostate cancer. Understanding these interactions is essential for developing targeted public health interventions, such as weight management programs tailored to high-risk populations. Further research is needed to clarify the causal pathways between BMI and prostate cancer risk, particularly to determine whether BMI can serve as a modifiable risk factor for prevention or early detection. Addressing this gap could also open new avenues for personalized medicine approaches, offering more precise recommendations to reduce prostate cancer burden globally.

## Methods

### Study design and reporting standards

This systematic review was conducted to evaluate the association between body mass index (BMI) and prostate cancer incidence and screening outcomes. No human participants were directly recruited, as this research involved secondary analysis of published literature only. The review followed the Preferred Reporting Items for Systematic Reviews and Meta-Analyses (PRISMA) guidelines. This review was not prospectively registered, and this is acknowledged as a limitation of the study.

### Literature search strategy

A comprehensive literature search was conducted using the PubMed/MEDLINE and Cochrane Library databases. The search included peer-reviewed articles published between 2004 and November 2025. The primary keyword search string applied was: “BMI AND prostate cancer AND screening.” The search strategy was designed to identify studies evaluating the relationship between BMI and prostate cancer incidence and screening-related outcomes.

### Eligibility criteria

Inclusion criteria:

- Studies examining prostate cancer in relation to BMI metrics
- Peer-reviewed publications
- Study designs including randomized controlled trials (RCTs), clinical trials, systematic reviews, and meta-analyses

Exclusion criteria:

- Case reports
- Editorials and letters
- Commentaries
- Studies without measurable outcomes related to prostate cancer or screening
- Non-peer-reviewed publications

### Study selection

**Figure 1:**
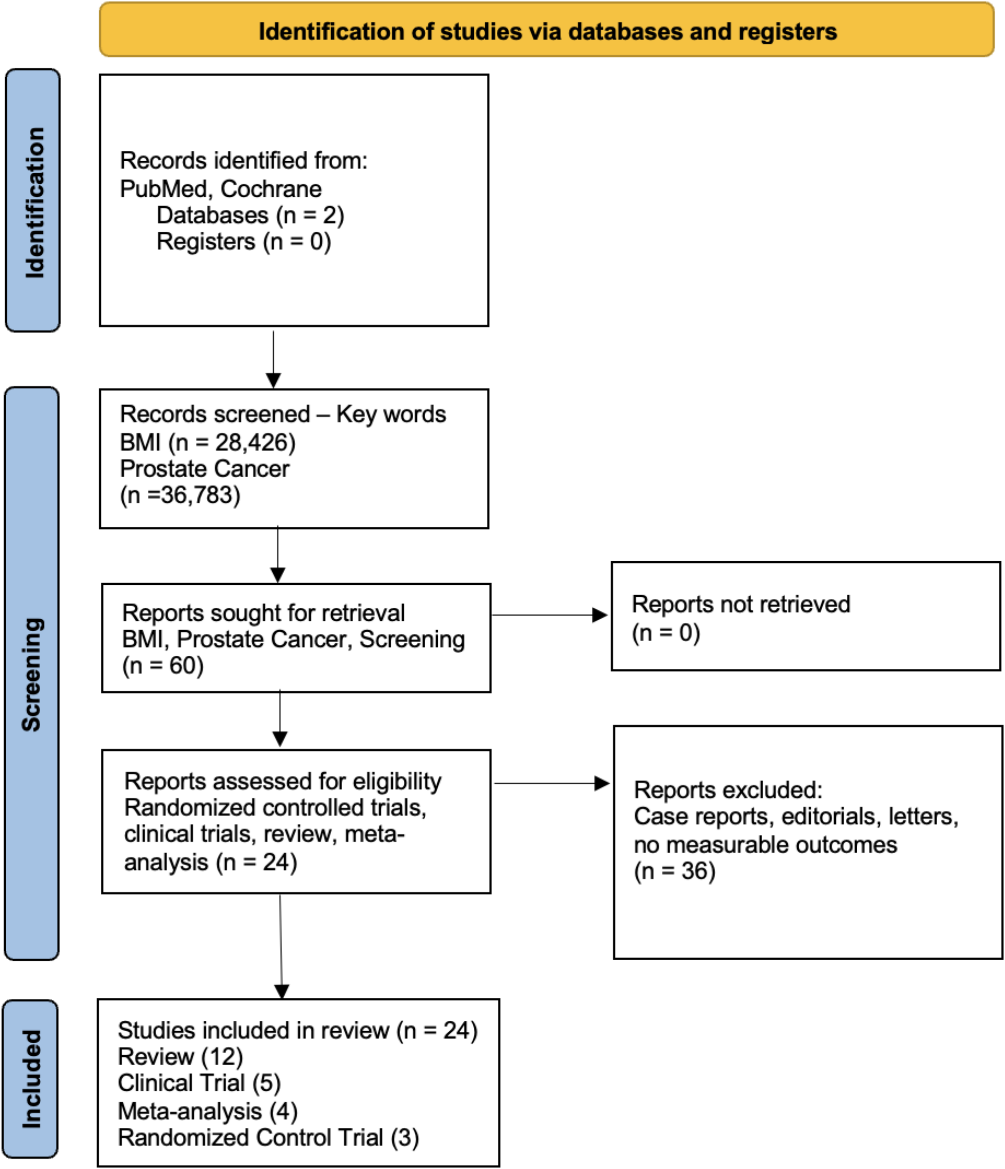
Study selection process and PRISMA flow chart.

All retrieved citations were imported into Zotero for reference management and duplicate removal. Titles and abstracts were screened for eligibility, followed by full-text review of potentially relevant articles. The study selection process was conducted independently by two reviewers. Discrepancies were resolved through discussion and consensus. The study selection process is presented in **Figure 1** (PRISMA Flow Diagram).

### Data extraction

Two independent reviewers conducted data extraction using a predefined standardized Excel spreadsheet. Extracted variables included:

- Author(s)
- Year of publication
- Study design
- Study population and methods
- Main findings

Zotero was used as a citation management system and for maintaining structured summary notes for each included study.

### Quality assessment and risk of bias evaluation

Methodological quality and risk of bias were assessed using an AMSTAR 2–based assessment framework. Core AMSTAR 2 domains were applied to evaluate methodological rigor, internal validity, and report quality across the included studies. The assessment focused on the following domains:

- Clarity of research objectives and inclusion criteria
- Adequacy and transparency of the search strategy
- Appropriateness of study selection and data extraction methods
- Consistency and reliability of outcome measurements
- Reporting completeness and methodological reproducibility

Each study was independently evaluated by two reviewers. Disagreements were resolved by consensus. Studies were categorized into overall confidence levels of high or low methodological quality. Methodological quality ratings of “high” were only included in the paper. Papers with “low” ratings were excluded.

### Data synthesis

A qualitative synthesis was conducted for all included studies. Due to heterogeneity in study design, population characteristics, outcome definitions, and measurement methods, formal quantitative meta-analysis was not performed. Methodological heterogeneity and risk-of-bias ratings were considered in interpreting the strength and consistency of evidence across studies.

### Study identification results

The initial search identified 28,426 studies using the keyword “BMI” and 36,783 studies using the keyword “prostate cancer.” A combined search using the keywords “BMI” and “prostate cancer” yielded 186 studies, and the full search string “BMI AND prostate cancer AND screening” yielded 60 studies.

After screening and eligibility assessment, 24 studies were included for direct analysis, and 36 studies were excluded based on predefined inclusion and exclusion criteria. No additional eligible studies were identified from the Cochrane database.

The final included studies comprised randomized controlled trials, clinical trials, and meta-analyses. The full selection process is illustrated in **Figure 1**.

## Results

Data extraction was performed by two independent reviewers using a standardized Excel spreadsheet to capture relevant information, including the author(s), publication year, study design, study population, methods, and main findings.

The initial keyword search for “BMI” yielded 28,426 studies. An additional 36,783 studies were retrieved using the keywords “Prostate Cancer.” Combining keywords, “BMI” and “Prostate Cancer” resulted in 186 studies. 60 articles were found with “BMI, Prostate Cancer, Screening”. Following a thorough screening process based on pre-established inclusion criteria, 36 studies were excluded. The study selection process is detailed in **Figure 1**. Consequently, 24 studies were selected for direct analysis which are shown in **Table 1**. These studies included randomized controlled trials (RCTs), clinical trials, reviews, and meta-analyses.

**Table 1:** Included articles (author/year, study design, population/methods, main findings).

Author(s), year	Study design	Study population/Methods	Main findings
Allott et al., 2013 (21)	Review	Literature review on articles published between 1991 and July 2012 using PubMed with the following terms: obesity, BMI, body mass index and prostate cancer risk, prostate cancer incidence, prostate cancer mortality, radical prostatectomy, androgen-deprivation therapy, external-beam radiation, brachytherapy, prostate cancer and quality of life, prostate cancer and active surveillance, in addition to obesity, BMI, body mass index and prostate cancer and insulin, insulin-like growth factor, androgen, estradiol, leptin, adiponectin, and IL-6.	Weight loss slows prostate cancer in animals but lacks human trial validation.
Barrington et al., 2015 (1)	Clinical trial	Prospective study of 3398 African American and 22,673 non-Hispanic white men who participated in the Selenium and Vitamin E Cancer Prevention Trial (2001–2011).	Obesity increases prostate cancer risk more in African Americans than in non-Hispanic whites, suggesting weight loss could reduce racial disparities.
Bayraktar et al., 2023 (9)	Clinical trial	Total of 908 men underwent biopsies with transrectal ultrasound, of which 492 (51.5%) had Metabolic Syndrome according to ATP III criteria.	Biomarkers such as CD169, neuropilin-1, cofilin-1, IL-17, STAT3, LIMK1, PSMA, AMACR, and others may aid in prognosis and serve as potential targets for immunotherapy or radionuclide therapy.
Cao and Giovannucci 2016 (7)	Review	Book chapter in Obesity and Cancer pp 137–153	Different molecular subtypes of prostate cancer may provide insights into its causes.
Efstathiou et al., 2007 (22)	Clinical trial	Between 1987 and 1992, 945 eligible men with prostate cancer were enrolled in a phase 3 trial (RTOG 85-31) and randomized to Radiation Therapy and immediate goserelin or Radiation Therapy alone followed by goserelin at recurrence. Height and weight data were available at baseline for 788 (83%) subjects.	Greater baseline BMI is independently associated with higher Prostate Cancer Specific Mortality in men with prostate cancer. Research is needed on whether weight loss after diagnosis improves outcomes and reduces cancer-specific mortality.
Grubb et al., 2009 (12)	Randomized control trial	38,349 men ages 55 to 74 years randomized in Prostate, Lung, Colorectal, and Ovarian (PLCO) Cancer Screening Trial received annual PSA and digital rectal examination screening, 28,380 had a baseline PSA, complete demographic information, and no prostate cancer diagnosis within 6 years from baseline	Lower PSA levels in obese men may result from hemodilution, affecting screening accuracy.
Harrison et al., 2020 (13)	Meta-analysis	Literature Review on PubMed and Embase for studies up until October 2, 2017, and obtained individual participant data from four studies. In total, 78 studies were identified for the association between BMI and prostate cancer, 21 for BMI and prostate cancer, and 35 for BMI and PSA.	BMI has little impact on prostate cancer risk, but higher BMI is linked to lower PSA levels.
Hu et al., 2014 (3)	Meta-analysis	Systematic review and dose-response meta-analysis of published studies from MEDLINE and EMBASE to determine the relationship between body mass index (BMI) and Biochemical Recurrence of prostate cancer; Total of 26 studies including 36,927 individuals were used.	Excess BMI correlates with cancer recurrence, highlighting the need for weight control to improve prognosis.
Kampman et al., 2012 (6)	Review	Current Nutrition Reports Article 2012 pages 30–36	Studies should explore how diet and exercise can change behavior to improve outcomes.
Karwacki et al., 2024 (20)	Meta-analysis	389,918 prostate cancer patients who had radical prostatectomy with final histopathology reporting lymphovascular invasion status.	Higher preoperative PSA, higher clinical T stage, and higher biopsy Gleason score were significantly associated with lymphovascular invasion, while BMI, age, and prostate volume showed no meaningful association.
Kielb et al., 2023 (10)	Review	PUBMED/Scopus database and gray literature to conduct a thorough search for original and review articles published up to December 2022 using the following terms: prostate cancer; biomarkers; IL-17; STAT3; NRP1; LIMK1; Cofilin-1; PSMA; AMACR; CD15; Appl1; Sortilin; Syndecan-1, and p63. 275 articles were selected.	Biomarkers could serve as targets for immunotherapy or radionuclide therapy in the future.
Lewis et al., 2010 (8)	Clinical trial	1,550 screen-detected prostate cancer cases, 1,815 age-matched controls with unrestricted PSA testing, and 1,175 low-PSA controls, evaluating whether the obesity-related FTO rs9939609 variant (associated with higher BMI) was linked to prostate cancer risk.	The FTO rs9939609 A allele—correlated with higher BMI—was weakly associated with lower overall and low-grade prostate cancer risk but showed a modest trend toward increased odds of high-grade versus low-grade disease among cases.
Mantzorou et al., 2017 (4)	Review	Literature Review to summarize the prognostic role of nutritional status, from Body Mass Index (BMI) and weight loss to nutrition screening tools and biochemical indices, in cancer patients.	Prospective research is needed on how nutrition and biochemical markers impact prognosis.
Muller et al., 2013 (16)	Randomized control trial	Secondary analysis of the Reduction by Dutasteride of prostate Cancer Events (REDUCE) trial, which was originally aimed at cancer risk reduction among high-risk men with a single negative prestudy biopsy.	Obesity increases prostate volume and reduces the effectiveness of drugs like dutasteride.
Murtola et al., 2018 (24)	Randomized control trial	Participants of the Finnish Randomized Study of Screening for Prostate Cancer (FinRSPC) were linked to laboratory database for information on glucose measurements since 1978. The data were available for 17,860 men.	High fasting glucose levels may increase the risk of prostate cancer.
O’Malley et al., 2006 (15)	Review	Literature review on current body of evidence	Proper timing of serum tests is crucial to determine if markers are causative or produced by cancer.
Renehan et al., 2010 (11)	Review	Literature review using sex- and population-specific risk estimates were determined for associations with BMI in a standardized meta-analysis for 20 cancer types	Prostate screening attenuates BMI associations when all prostate cancers are considered together. BMI is differentially associated with different histological subtypes within the same cancer group.
Shi et al., 2021 (18)	Review	Literature review using PubMed, EMBASE, and Cochrane Library databases for relevant literature and subjected the resulting articles to meta-analysis.	Obesity has a weak link to prostate and bladder cancers, but the evidence is limited.
Shi et al., 2024 (19)	Meta-analysis	Literature review using PubMed and other databases up to July 2023 using the keywords related to ‘risk’, ‘cancer’, ‘weight’, ‘overweight’, and ‘obesity’	Obesity may be linked to a reduced risk of prostate cancer, though the relationship is unclear.
Skolarus et al., 2007 (23)	Review	Literature review on current literature regarding body mass index (BMI) and its relationship with various clinical aspects of prostate cancer, including its incidence, screening, diagnosis and treatment.	Treating prostate cancer in obese patients is difficult, with unclear data on treatment outcomes.
Stewart and Freedland 2011 (2)	Review	PubMed search was performed using key words related to incidence, treatment, obesity, prostate, kidney, and bladder cancer.	Obesity appears to promote an increased risk of prostate cancer.
Tarantino et al., 2021 (14)	Review	Reviewed the medical records of 833 prostate cancer patients undergoing radical prostatectomy.	PSA correlates with tobacco use, supporting its use for screening in smokers.
Wang Y et al., 2025 (17)	Review	10,842 men undergoing surgery for presumed benign prostatic hyperplasia, of whom 957 were later found to have incidental prostate cancer on final histopathology.	Higher BMI, older age, higher pre-PSA and pre-PSAD, smaller resected prostate weight, no 5-ARI use, positive family history, and abnormal DRE/TRUS findings were significantly associated with increased odds of incidental prostate cancer, while baseline prostate volume, smoking, hypertension, diabetes, dyslipidemia, and MRI findings were not.
Zilli et al., 2011 (5)	Clinical trial	112 eligible patients with intermediate-risk prostate cancer	Higher abdominal fat and BMI are linked to earlier diagnosis and larger prostate volume.

The main findings of the selected studies were systematically summarized to evaluate the influence of BMI on prostate cancer screening outcomes. Data extraction identified a complex and multifaceted relationship between Body Mass Index (BMI) and prostate cancer incidence. This relationship reflects a combination of biological, genetic, and environmental influences that shape disease risk, sometimes in contradictory ways. While higher BMI is generally associated with an increased risk of prostate cancer and worse clinical outcomes, the connection extends beyond a straightforward positive correlation (2). Understanding these complexities is crucial for tailoring effective public health strategies to reduce prostate cancer risk.

**Figure 2:**
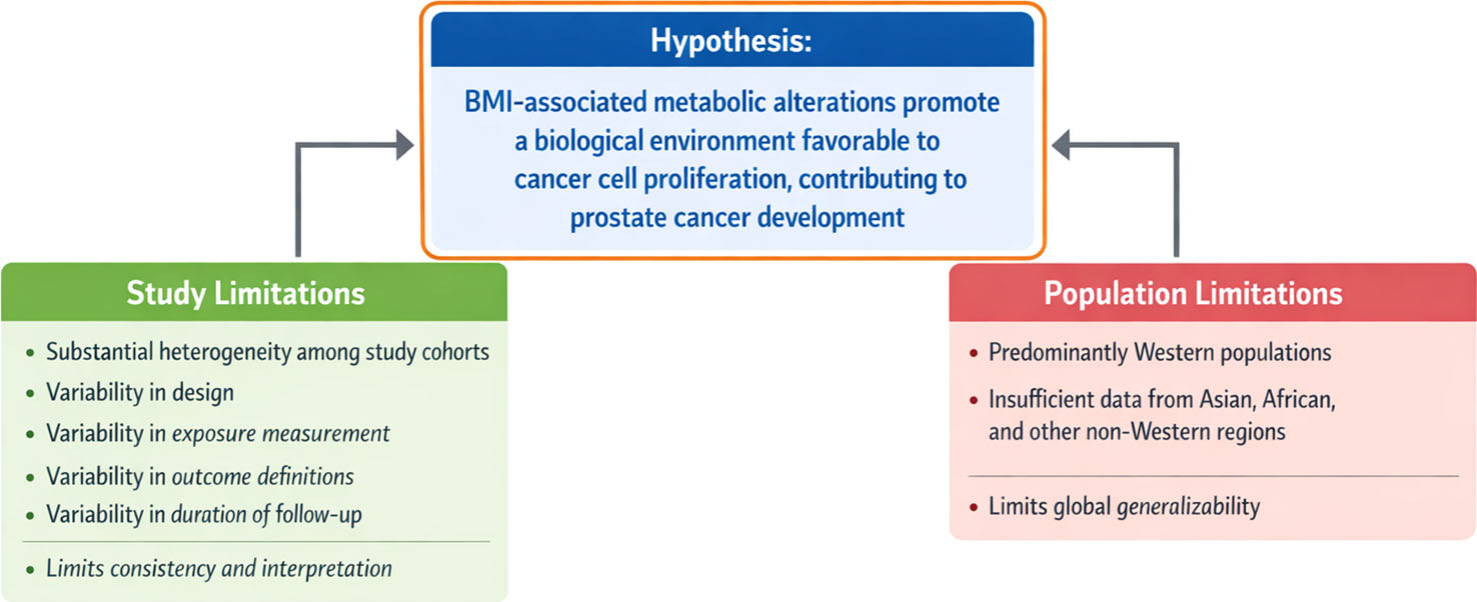
.

Although 24 studies met the inclusion criteria for direct analysis, pooling data into a meta-analysis was deemed inappropriate due to significant heterogeneity among the studies. Heterogeneity arose from clinical differences in BMI definitions and measurements, outcome variability across endpoints and follow-up durations, diverse study designs, and inconsistent reporting of results shown in **Figure 2**. While no formal quantitative analysis (e.g., subgroup analyses) was performed, these differences likely affect the consistency and generalizability of findings. Consequently, a meta-analysis was not feasible, and a structured narrative synthesis was used to integrate results while preserving methodological rigor.

### Biological mechanisms

Several potential biological mechanisms have been proposed to explain the link between BMI and prostate cancer. Obesity is often associated with metabolic changes that may influence cancer development. Several findings align with our hypothesis that obesity-related metabolic changes create a conducive environment for cancer cell proliferation.

Adipose tissue, mainly visceral fat, is metabolically active and secretes various hormones and cytokines that may promote tumor proliferation. Abdominal adiposity and high BMI are associated with younger age at diagnosis and greater prostate volume (Zilli et al., 2011) (5). Further research is needed and should be directed toward investigating diet and physical activity to change health behavior (Kampman et al., 2012) (6).

Biological mechanisms supporting the correlation are currently under investigation, but may involve insulin and IGF-1, sex steroid hormones, and alterations in metabolism. There is some data that suggests molecular sub-types of prostate cancer may offer insights into etiology (Cao et al., 2016) (7).

The relationship between BMI and prostate cancer also extends to genetic and molecular pathways. A genetic association study of the obesity-related FTO rs9939609 variant found that the A allele, which is strongly linked to an increased risk of obesity and higher body mass index (BMI), was linked to a lower incidence of prostate cancer overall and of low-grade tumors (Gleason <7) (Lewis et al., 2010) (8). In contrast, when the analysis was restricted to men who had already been diagnosed with prostate cancer, the same allele was associated with slightly higher odds of having high-grade rather than low-grade disease (Gleason ≥7), despite not increasing the overall incidence of high-grade cancer compared with controls (8). These findings show that the genetic influence on prostate cancer incidence differs from its influence on tumor grade at diagnosis.

Biomarkers might represent another prognostic factor, as discussed in many studies. Several histopathological biomarkers (particularly CD169 macrophages, neuropilin-1, cofilin-1, interleukin-17, signal transducer and activator of transcription protein 3 (STAT3), LIM domain kinase 1 (LIMK1), CD15, AMACR, prostate-specific membrane antigen (PSMA), Appl1, Sortilin, Syndecan-1, and p63) may be important in the prognosis and treatment of prostate cancer patients (Bayraktar et al., 2023) (9). The potential use of histopathological markers as a target for novel immunotherapeutic drugs or targeted radionuclide therapy, may be used as adjuvant therapy in the future (Kielb et al., 2023) (10).

In a study by Renehan et al., 2010, prostate screening can attenuate the association between BMI and prostate cancer because higher BMI is linked to a lower likelihood of a positive screening result (high PSA) (11). In fact, one study found an inverse relationship between PSA concentration and BMI which may be explained by a hemodilution effect, diluting the blood to maintain blood volume without a transfusion (Grubb et al., 2009) (12).

Current evidence shows little to no association between BMI and the overall risk of prostate cancer. Instead, there is strong evidence for an inverse, non-linear relationship between BMI and PSA levels (Harrison et al., 2020) (13). In patients who have undergone treatment with surgery or radiation, a rise in PSA may be incidental rather than clinically meaningful. One population in which PSA screening may provide added value is smokers: tobacco consumption has been correlated with elevated PSA levels, possibly due to hormonal or genetic influences (Tarantino et al., 2021) (14). Continued research is needed to clarify the biological mechanisms linking BMI and prostate cancer and to identify potential targets for preventive or therapeutic intervention (O’Malley et al., 2006) (15).

### Environmental mediators

The association between BMI and prostate cancer incidence appears to vary across demographic and environmental factors. Age-related differences have been noted in several studies. In a randomized controlled trial of 8,122 men, obesity was associated with greater prostate volume growth and reduced prostate volume response to dutasteride therapy, indicating that BMI may modify treatment response (Muller et al., 2013) (16).

Racial and ethnic variation has also been reported. In a clinical trial, obesity showed a stronger association with prostate cancer risk among African American men compared with non-Hispanic white men, suggesting differential risk patterns across populations (Barrington et al., 2015) (1). The specific contribution of BMI to these disparities remains uncertain.

Socioeconomic status may further influence this relationship. Lower income and education levels are associated with higher BMI and higher rates of chronic diseases, including some cancers (2). These patterns indicate that socioeconomic factors may function as mediators between BMI and prostate cancer risk, though the mechanisms remain incompletely defined.

### BMI and prostate cancer incidence

Several epidemiological studies have investigated the correlation between BMI and prostate cancer incidence. While some studies have reported a positive correlation between BMI and prostate cancer risk, others have found inconclusive or even inverse associations. Similarly, a recent meta-analysis of 10,842 men undergoing surgery for presumed benign prostatic hyperplasia reported an incidental prostate cancer (IPCa) rate of 8.83%. Higher BMI was significantly associated with IPCa, shown by a pooled standardized mean difference of 0.23 (95% CI: 0.10–0.35; P < 0.001), with no heterogeneity across studies. This association remained consistent across surgical techniques and study settings, indicating that men with higher BMI are more likely to have IPCa detected during surgery (Wang Y et al., 2025) (17).

Conversely, contradictory findings have also been reported in the literature. Only a weak association between overweight and obesity with prostate cancers was found, but due to the low quality of included systematic reviews, the results need to be interpreted with caution (Shi et al., 2021) (18). Interestingly, a recent metanalysis found that when compared to underweight or normal weight, overweight or obesity was associated with a decreased risk of prostate cancer (Shi et al., 2024) (19). A recent systematic review including 389,997 men undergoing radical prostatectomy showed that lymphovascular invasion was present in 8.7% of patients, with the strongest preoperative predictors being higher PSA, clinical T3 stage and biopsy Gleason score ≥8 (Karwacki et al., 2024) (20). Age, BMI and prostate volume did not show meaningful correlations with lymphovascular invasion (20).

These inconsistencies highlight the complex nature of the relationship between BMI and prostate cancer, which may vary depending on factors such as study population, interventional study design, and the prognosis of the disease.

### BMI and prostate cancer survival outcomes

A 2014 systematic review and dose–response meta-analysis by Hu et al. demonstrated a positive correlation between higher BMI and biochemical occurrence of prostate cancer. The authors suggested that weight control may influence prostate cancer outcomes. Evidence from animal studies also indicates that weight loss after diagnosis may alter disease progression, but human data remain limited (Allott et al., 2013) (Efstathiou et al., 2007) (21, 22).

Data on oncologic and functional outcomes remain inconsistent. Some studies report lower BMI as an independent prognostic factor for reduced survival, while others find no association between BMI and survival after diagnosis (Skolarus et al., 2007) (23). Additional prospective and interventional studies are needed to clarify the prognostic value of nutritional assessment tools and biochemical indicators of nutritional status (Mantzorou et al., 2017) (4).

## Discussion

The association between BMI and prostate cancer reflects a complex set of biological and environmental mechanisms that extend beyond simple correlations. Elevated BMI has been consistently linked with higher risk of disease, poorer clinical outcomes, and altered responses to diagnostic and therapeutic interventions (2). These associations suggest the presence of underlying causal pathways through which excess adiposity may influence both the development and progression of prostate cancer.

One potential mechanism involves the hormonal and metabolic effects of obesity. Increased adipose tissue promotes peripheral conversion of androgens, alters insulin and insulin-like growth factor signaling, and contributes to systemic inflammation, which may be drivers of tumor initiation and progression (21). Elevated fasting glucose and other markers of metabolic dysfunction have also been associated with prostate cancer risk, reinforcing the idea that metabolic disturbances commonly observed in obesity may contribute to carcinogenesis (Murtola et al., 2018) (24).

Obesity may also influence prostate cancer incidence through its effect on screening performance. Hemodilution in men with higher BMI lowers circulating PSA concentrations, which can mask early-stage disease and delay detection (12). This mechanism offers a possible causal explanation for the observed association between elevated BMI and disease at diagnosis (Stewart and Freedland, 2011) (2). It also suggests that obesity does not merely correlate with screening disparities but may directly influence diagnostic sensitivity through physiological pathways.

Once prostate cancer is present, obesity related biological changes appear to shape disease behavior. Chronic low-grade inflammation altered cytokine signaling, and dysregulation of immune cell function may contribute to various tumor phenotypes (2). Increased prostate volume in men with elevated BMI may further reduce the efficacy of some treatments, including agents that target prostatic tissue (7, 9). These pathways suggest that obesity can modify the tumor microenvironment and influence treatment response.

Emerging biomarker research provides additional insights into the potential mechanistic links between adiposity and prostate cancer progression. Molecules such as IL-17, STAT3, PSMA, and AMACR are involved in pathways related to immune activation, metabolic signaling, and cellular proliferation (9, 10). Their expression in patients with higher BMI raises the possibility that excess adiposity may drive changes in tumor biology that alter therapeutic susceptibility.

Lifestyle related factors associated with obesity may also contribute indirectly to disease outcomes. Diet, physical inactivity, and metabolic stress intersect with inflammatory and hormonal pathways implicated in carcinogenesis (6). These relationships suggest that the link between BMI and prostate cancer may be partially mediated by modifiable behaviors that shape metabolic and inflammatory profiles.

### Future directions

Future research should more clearly delineate the complex and multifactorial relationship between body mass index and prostate cancer. Longitudinal studies that characterize the timing, duration, and severity of obesity are needed to clarify how distinct obesity trajectories influence prostate cancer development and progression. A priority for upcoming work is the identification of metabolic, hormonal, and genetic markers that confer increased susceptibility to prostate cancer among individuals with excess adiposity. Such biomarkers would facilitate the development of more refined screening algorithms and support a more individualized approach to prevention.

The design of targeted interventions represents another important area for investigation. Weight management programs specifically tailored to men at elevated risk of prostate cancer may contribute to reductions in disease incidence and improvements in prognosis. In parallel, combined strategies that integrate lifestyle modification with pharmacologic therapies aimed at mitigating obesity-related metabolic dysfunction warrant further examination. These interventions should be subjected to rigorous evaluation to determine their long-term effectiveness in both preventing the onset of prostate cancer and improving outcomes among patients with established disease.

The current body of evidence supports a multifactorial framework in which obesity influences prostate cancer biology through interconnected physiological pathways. These include metabolic dysregulation, hormonal alterations, chronic inflammation, modifications of the tumor microenvironment, and reduced sensitivity of PSA-based screening.

A clearer understanding of these pathways has direct clinical implications. Incorporating body mass index and broader metabolic risk profiles into clinical risk assessment, accounting for diminished PSA sensitivity in individuals with elevated adiposity and recognizing the biological impact of excess adipose tissue during treatment planning may enhance the precision of clinical decision-making. Future studies should prioritize the elucidation of causal mechanisms and the identification of biomarkers that clarify how adiposity shapes disease trajectory with the aim of informing targeted preventive and therapeutic strategies.

### Conclusion

A comprehensive understanding of the role of body mass index and metabolic health in prostate cancer development is essential for advancing both prevention and clinical management. Current evidence indicates that excess adiposity interacts with hormonal, inflammatory, and metabolic pathways that influence tumor initiation and progression. For clinicians, these findings underscore the importance of integrating metabolic risk profiling into prostate cancer assessment, particularly for individuals with central obesity, insulin resistance, or other cardiometabolic abnormalities. From a population health perspective, interventions that promote weight management, physical activity, and improved metabolic regulation may help reduce prostate cancer incidence and support more favorable long-term outcomes.

Interpretation of existing research is limited by substantial heterogeneity among studies, including variability in design, exposure measurement, outcome definitions, and duration of follow-up. The included studies are predominantly from Western populations with insufficient representation of data from Asian, African, and other non-Western regions. This limits the generalizability of the conclusions to global populations. These limitations highlight the need for rigorous longitudinal studies that incorporate detailed body composition metrics, metabolic biomarkers, and molecular profiling. Future research will be essential for clarifying causal pathways, identifying high-risk subgroups, and guiding the development of targeted interventions with the potential to more effectively reduce prostate cancer morbidity and mortality.

## Data Availability

The original contributions presented in the study are included in the article/supplementary material. Further inquiries can be directed to the corresponding author.
